# Measurement of Internal Implantation Strains in Analogue Bone Using DVC

**DOI:** 10.3390/ma13184050

**Published:** 2020-09-12

**Authors:** Alexander Marter, Charles Burson-Thomas, Alexander Dickinson, Kathryn Rankin, Mark Mavrogordato, Fabrice Pierron, Martin Browne

**Affiliations:** 1 Bioengineering Science Research Group, School of Engineering, University of Southampton, Southampton SO17 1BJ, UK; a.d.marter@soton.ac.uk (A.M.); c.bursonthomas@soton.ac.uk (C.B.-T.); alex.dickinson@soton.ac.uk (A.D.); 2 Institute for Life Sciences, University of Southampton, Southampton SO17 1BJ, UK; 3 µVIS X-ray Imaging Centre, University of Southampton, Southampton SO17 1BJ, UK; k.rankin@soton.ac.uk (K.R.); mnm100@soton.ac.uk (M.M.); 4 Materials Research Group, School of Engineering, University of Southampton, Southampton SO17 1BJ, UK; f.pierron@soton.ac.uk

**Keywords:** cementless implant, Digital Volume Correlation (DVC), micro-CT, FE modelling, analogue bone

## Abstract

The survivorship of cementless orthopaedic implants may be related to their initial stability; insufficient press-fit can lead to excessive micromotion between the implant and bone, joint pain, and surgical revision. However, too much interference between implant and bone can produce excessive strains and damage the bone, which also compromises stability. An understanding of the nature and mechanisms of strain generation during implantation would therefore be valuable. Previous measurements of implantation strain have been limited to local discrete or surface measurements. In this work, we devise a Digital Volume Correlation (DVC) methodology to measure the implantation strain throughout the volume. A simplified implant model was implanted into analogue bone media using a customised loading rig, and a micro-CT protocol optimised to minimise artefacts due to the presence of the implant. The measured strains were interpreted by FE modelling of the displacement-controlled implantation, using a bilinear elastoplastic constitutive model for the analogue bone. The coefficient of friction between the implant and bone was determined using the experimental measurements of the reaction force. Large strains at the interface between the analogue bone and implant produced localised deterioration of the correlation coefficient, compromising the ability to measure strains in this region. Following correlation coefficient thresholding (removing strains with a coefficient less than 0.9), the observed strain patterns were similar between the DVC and FE. However, the magnitude of FE strains was approximately double those measured experimentally. This difference suggests the need for improvements in the interface failure model, for example, to account for localised buckling of the cellular analogue bone structure. A further recommendation from this work is that future DVC experiments involving similar geometries and structures should employ a subvolume size of 0.97 mm as a starting point.

## 1. Introduction

Long-term aseptic loosening is a primary failure mode of orthopaedic implants [1]; the quality of fixation plays a crucial role in arthroplasty outcomes. The number of implantations into younger, heavier, and more active patients is increasing. Cementless implants have been developed to attempt to meet the demands of such patients. These implants achieve long-term fixation by bone integration (osseointegration) with the implant’s surface. However, this takes time: retrieval of failed implants and animal studies have shown long-term osseointegration typically takes between 4 and 12 weeks [2]. Thus, immediate implant–bone stability (upon implantation) is required to facilitate the gradual process of osseointegration [2]. During surgery, bone is removed by either reaming or broaching to produce a cavity. The relative difference in the size of the cavity to the dimensions of the implant generates stress in the bone, resulting in friction at the implant–bone interface, that generates the initial fixation. The ‘quality’ of the interference fit is influenced by the level of interference, damage caused by implantation, implant design (shape and surface roughness), and bone quality [3]. A better understanding of periprosthetic bone strain generated upon implantation would support implant development in several ways: reduce the incidence of post-implantation fractures, improve osseointegration via better control of stress-shielding effects, and reduce micromotion at the implant–bone interface by optimising the local deformation.

Strain gauges and extensometers have been extensively used in orthopaedic research to aid optimisation of implant designs and surgical techniques [4,5,6]. They offer simple and reliable means to accurately measure strains at discrete locations on a surface. Whilst very accurate, their usefulness is limited by a number of shortcomings, most notably that they provide discrete data at accessible locations, and do not provide full-field strain. Digital Image Correlation (DIC) is a non-contact method of measuring full-field surface displacements and strains. Measurements are generated by tracking changes to an applied or naturally occurring random pattern on a specimen’s surface via the use of a digital camera whilst the specimen is loaded. DIC has become a popular measurement technique for biomechanical applications and has been used for studies conducted on synthetic bone [7,8,9], soft tissues [10], micro-level bone properties [11], and whole bones [12]. However, the region of greatest interest during cementless implantation is the implant–bone interface, and volume in close proximity, for which strain measurement by DIC is infeasible.

First described in 1999 by Bay et al. [13], Digital Volume Correlation (DVC) is an extension of DIC using 3D imaging. Like DIC, DVC measures displacement by tracking a naturally occurring texture or artificially generated pattern (e.g. seeded polymers, sintered materials). However, this tracking occurs within a body, from its reference to a deformed state, enabling calculation of the volumetric strain field. DVC has already enabled several biomechanical findings that would not have been possible by surface or discrete strain measurement methods alone. For example, when investigating bone–cement interfaces, Tozzi et al. [14] were able to identify regions of residual strain where failure was not visually apparent. The strain results also suggested that higher bone strength increased the risk of failure at the interface. In another study, where DVC was used to investigate porcine vertebrae, it was possible to identify regions of high local-strain, which enabled failure sites to be determined before becoming apparent by visual inspection of scan data or surface measurement [15]. A follow-on study, with prophylactic augmented vertebrae, demonstrated lower strains were experienced within the stiffer cement region, resulting in strain concentrations where cement did not permeate into bone and subsequent localised failure [16]. 

A small number of DVC studies have been conducted on implanted bone constructs. However, these have either analysed strains generated during screw pull-out [17,18,19,20], or investigated the localised micro-motion [21,22]. The strain generated by the implantation of cementless implants is currently unexplored by full-field methods. In this work, we have devised a DVC methodology to measure the strain generated from implantation, throughout the volume. In addition, a corresponding FE model was developed to aid the interpretation of the measurements. The overall objective was to better understand the nature and mechanisms of strain generation during implantation.

## 2. Materials and Methods 

### 2.1. Analogue Bone

Ideally, testing of an implanted construct would be performed on the most representative model—human bone. However, the cost, availability, and ethical considerations of employing human bone often make this prohibitive. As an alternative, analogue bone has been extensively used in orthopaedic research for mechanical testing of implants. These commercially manufactured materials are available as whole bone-like structures and blocks of synthetic foam. They do not replicate the highly anisotropic mechanical properties of natural bone. However, they avoid the considerable inter-specimen variability in mechanical properties of natural bone which often limits a study’s external validity. Therefore, in this study, experiments were conducted with analogue bone.

**Figure 1:**
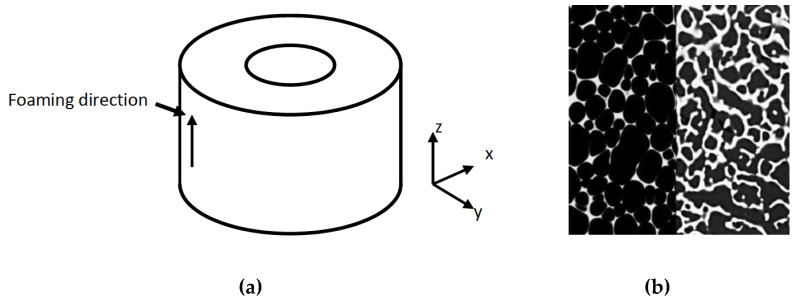
Analogue bone geometry and cellular structure: (**a**) the cylindrical piece of cellular polyurethane foam (density 320 kg⋅m^−3^) had an inner diameter of 15 mm, outer diameter of 45 mm, and height 40 mm; (**b**) closed-cell structure of polyurethane foam (**a**) compared with open-cell structure of bovine cancellous bone (**b**).

A cylindrical specimen of 320 kg⋅m^−3^ cellular polyurethane foam (Sawbones, Malmö, Sweden)—the analogue bone—was manufactured with an outer diameter of 45 mm, inner diameter of 15 mm, and height 40 mm using a milling machine. These dimensions were selected in order for the specimen to fill the field of view at the resolution of testing (~30 μm). The specimen was oriented as shown in Figure 1, such that the foaming direction was parallel with the reamed cavity.

The mechanical behaviour of cellular polyurethane foam has been assessed in relation to natural bone for both static [23,24,25,26,27,28] and dynamic [29] loading. It exhibits mechanical properties akin to the lower range of cancellous bone (depending on foam density); a similar stress–strain curve is observed, exhibiting near-linear elasticity followed by a plateau region and then consolidation.

### 2.2. Customised Loading Rig

In order to recreate the process of implantation within the Computed Tomography (CT) scanner for subsequent DVC analysis, a customised loading rig was developed, along with a simplified implant. A hollow aluminium pin with a nominal external diameter of 15.5 mm and hemispherical tip was lubricated with petroleum jelly and inserted into the central cavity of the analogue bone. The implant interference level was chosen to match the clinically recommended interference of 0.5 mm [30]. The geometry of the implant was selected to be axisymmetric in order to allow for circumferential averaging of the strain measurements, and thereby account for any small eccentricity or misalignment between the implant and cavity. The wall thickness of the aluminium pin was sufficiently large that it can be considered rigid in comparison to the analogue bone. 

**Figure 2:**
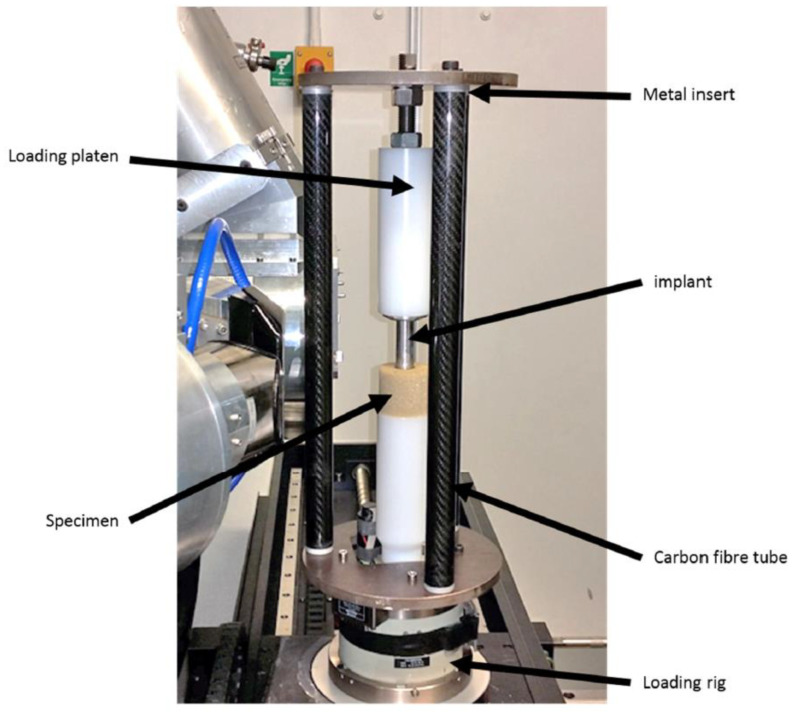
Customised loading rig to recreate process of implantation within micro-CT scanner. An aluminium stem (axisymmetric, non-tapered) was used as a simplified implant. Three carbon fibre tubes were used to minimise rigid body motion. Loading was conducted in displacement-control.

A support structure was required to constrain the assembly and allow application of compressive loading. This needed to be constructed from a material that would not substantially attenuate X-rays. Three carbon fibre tubes were incorporated in the loading rig to provide the necessary support—as shown in Figure 2. Aluminium inserts were glued into the tube ends to allow bolting to the top and bottom plates. Each aluminium insert extended 35 mm into the tube ends to ensure no interference with the field of view/X-ray beam, whilst still providing sufficient stability.

A single-axis actuator (Deben UK Ltd., Bury St Edmunds, UK), with a 1 kN capacity and 10 mm travel, was used to generate the displacement-controlled loading. The rig was initially positioned so that the analogue bone was towards the base of the field of view, and then displaced upwards relative to the stationary implant. Scans were then carried out at three displacement increments of 3 mm following a 20 min wait between scans to allow for viscoelastic stress relaxation in the analogue bone material. Each load step was held at constant displacement, with load measured throughout the test.

### 2.3. DVC Approach

In order to measure the implantation strain throughout the volume using DVC, micro-focus X-ray CT (μCT) was used first to image the internal volumetric structure of the analogue bone at each load step. μCT scans were acquired using the 225 kVp X-ray source in a custom 225/450kVp Nikon/X-tek HUTCH μCT scanner (Nikon/Metris, Tring, UK; μ-VIS centre, Southampton, UK) with a Perkin Elmer XRD 1621 CN03 HS detector (PerkinElmer Optoelectronics, Germany). The loading rig was positioned on the rotate stage with a source-to-object distance of 115.7 mm and a source-to-detector distance of 902.2 mm. The X-ray beam conditions were set with a peak voltage of 100 kVp and 633 μA current, with 1.6 mm aluminium filtration of the beam, using a rotating anode X-ray target head assembly. This filtered out lower energy photons from the polychromatic beam to reduce CT artefacts, including beam-hardening and scatter around the aluminium implant. A total of 3142 projections images were acquired with a 134 ms exposure time through 360° rotation, averaging 8 frames per projection to improve the signal-to-noise ratio. The projection images were reconstructed in 32-bit float volumes using CT-Pro3D software (Nikon, Tring, UK), which uses a filtered back-projection reconstruction method. A voxel (cubic pixel) size of 25.6 μm was achieved such that visualisation of pore walls was possible. Following reconstruction, volumes were downsampled to 8-bit using ImageJ [31] to reduce data processing time, which has been shown not to degrade the quality of the DVC results [32].

DVC analysis was performed to measure the displacement and strain fields of (i) each scan relative to the original scan and (ii) sequentially between Scans 1 and 2, and 2 and 3. In order to determine an optimal subvolume size and overlap, a noise and virtual displacement study was conducted. The noise study consisted of three activities: (1) repeat scans, (2) a translation of 5 voxels, and (3) a magnification change of 0.5%. To further test strain measurement accuracy in the absence of errors induced from changing the scan magnification (i.e. partial volume effects), DVC was performed between virtual deformations. A uniform axial compressive strain of 0.55%, 2%, and 10% was applied to the second repeat scan using ImageJ. Bicubic interpolation was used to perform the compression ‘virtually’, with each end being padded with blank slices such that the volume size remained consistent. The results of this are documented later, in Section 3.1.

A local DVC approach was selected as it is more computationally efficient than the global approach, with minor differences in accuracy [33]. DVC analysis was performed using DaVis software (Lavision, Göttingen, Germany). Two proprietary correlation algorithms are available in DaVis to correlate between subvolumes: direct correlation and Fast Fourier Transform (FFT). Direct correlation was selected over FFT, as it has been shown to be considerably more accurate [33]. Whole-voxel rigid body motion of each loaded scan was corrected prior to each analysis, using ImageJ. Fourth-order spline interpolation was used to improve the accuracy of sub-voxel displacement measurement. Displacement uncertainties were decreased by using a multi-pass approach, three in total, where large subvolumes are first used to inform subsequent passes [34]. The subvolume size of each pass was a multiple of the final pass: the first pass being three times the final subvolume size, and the second being twice the final subvolume. To account for any bulk greyscale scale changes or offset between scans, a direct zero-mean normalised cross-correlation criterion was used. 

Strains were calculated by centred finite differences without any additional smoothing [32]. These cartesian strains were then transformed into cylindrical coordinates. The strains were then averaged circumferentially about subvolume-sized radial increments. To reduce error from excessive deformation deteriorating correlation accuracy, the measurements were filtered to remove points with correlation coefficient values of less than 0.9 [32]. Finally, the data was cropped to the nominal internal and external diameters from the top of the block.

### 2.4. FE Modelling

**Figure 3:**
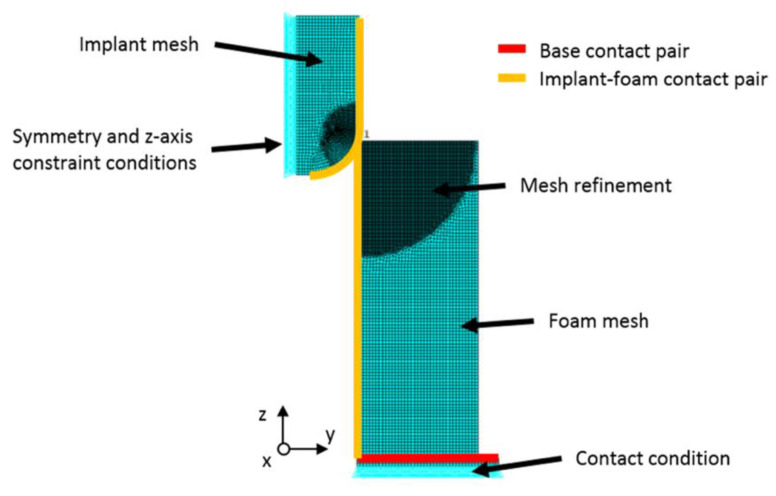
Axisymmetric Finite Element (FE) model of the experimental set-up to interpret results. Mesh refined close to the region of contact. Different boundary conditions used for the implant and analogue bone.

To better interpret the experimental results, a corresponding Finite Element (FE) analysis was performed. The software package ANSYS (Canonsburg, PA, USA) was used to conduct the displacement-controlled, implicit FE simulations. The rotational symmetry of the experimental setup allowed a 2D-axisymmetric model to be used, as shown in Figure 3. This reduced computational time and allowed a substantially higher mesh density than would be feasible in a 3D model, with local refinement close to the region of contact.

The compliance of the rig was excluded from the analysis by measuring implant insertion distance from each scan and applying this as an axial displacement to the implant. The friction coefficient was tuned by iteratively testing friction coefficients and comparing the model’s predicted reaction force to the experimental value. The analogue bone material was modelled as a continuum. Table 1 shows the magnitudes of the elastic moduli and Poisson’s ratios employed, using a transverse isotropy (orthotropic) material model. Shear modulus in the *x*–*z* and *y*–*z* planes were estimated from the results of Hamilton et al. [35]. Shear in the *x*–*y* plane was calculated from the relation between transverse modulus and Poisson’s ratio:

(1) $$G_{xy}=\frac{E_{x}}{2\left(1+\nu_{xy}\right)}.$$

A bilinear plasticity constitutive model was applied, where the Young’s modulus was reduced by three orders of magnitude once the yield strength of 5.4 MPa was reached. This reduction was chosen to simulate modulus tending to zero in the material’s crushed foam plateau region (as described in Section 2.1, [23,24,25,26,27,28,29]), whilst retaining numerical stability.

Strain results were exported to MATLAB for comparison with DVC results. To have a consistent spatial resolution, elemental FE strain results were averaged over volumes corresponding with the DVC subvolume size. As DVC was performed between Scan 0 and each implantation step, FE results of the starting position (reference) scan were subtracted from each displacement step.

## 3. Results

### 3.1. Noise and Virtual Displacement Study

**Figure 4:**
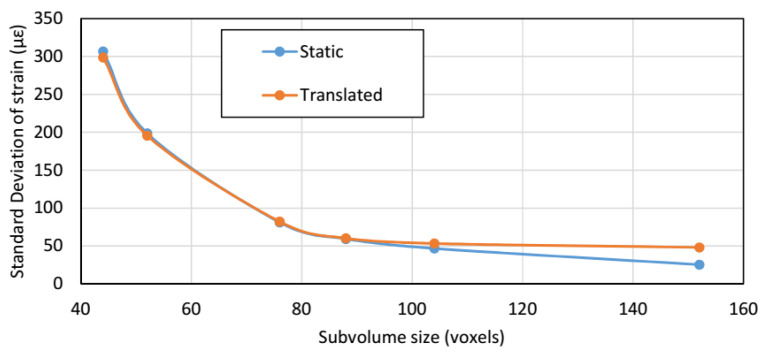
Influence of subvolume size on precision error between static and translated and scans.

**Figure 5:**
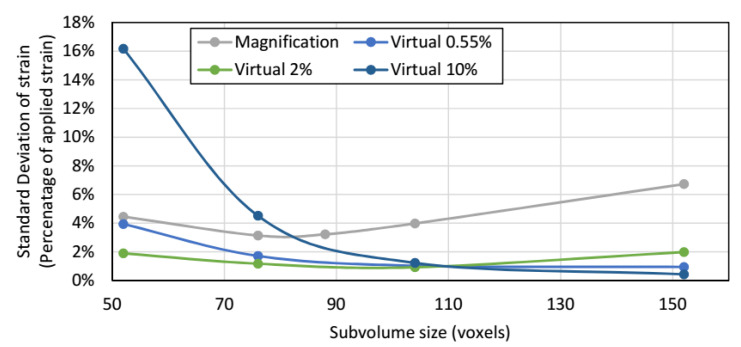
Influence of subvolume size on precision error between virtually strained and magnification change analyses.

Strain accuracy (bias; mean strain) of static and translated scans was less than 10 με for all subvolume sizes; the DVC calculated translation was 2% higher than the applied stage displacement. The strain accuracy of the magnification change was within 5% of that calculated from differences between scan resolutions. As shown in Figure 4, precision errors (standard deviation of strain) were near-identical for the static and translated tests, with translated scan errors being slightly higher at larger subvolume sizes. Figure 5 shows the precision errors resulting from the magnification change and virtual displacements. Precision error for the magnification change scans plateaued at the central subvolume size. The lowest accuracy error for the virtual strains was found at 2% applied virtual strain, whilst the 10% virtual strain had the largest error. The noise study and analysis using virtual displacements suggested a subvolume size of 76 voxels and an overlap of 50% would be appropriate, to give the optimal balance of low noise and high spatial resolution (25.6 um × 76 × 50% = 0.972 mm). 

### 3.2. Implantation Force and Friction from Modelling

Table 2 shows the measured reaction force and coefficient of friction used in the model, for each load step. The coefficient of friction was determined iteratively such that the FE simulation reaction force (at each step) was the same as that measured. The average achieved implantation was 2.9 mm per step.

### 3.3. Localised Crushing

**Figure 6:**
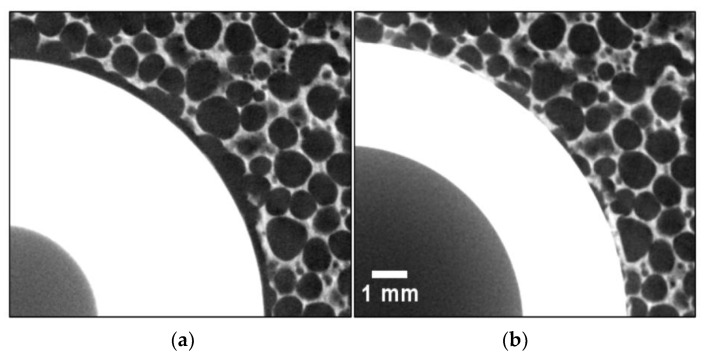
Localised crushing of foam struts: (**a**) before implantation; (**b**) after implantation. Note, the scale is the same for both images.

Inspection of implanted scans showed failure of the foam struts at the interface between the implant and analogue bone. Figure 6 shows an example of localised crushing in the cellular polyurethane foam.

### 3.4. Internal Implantation Strains

#### 3.4.1. Radial Direction

**Figure 7:**
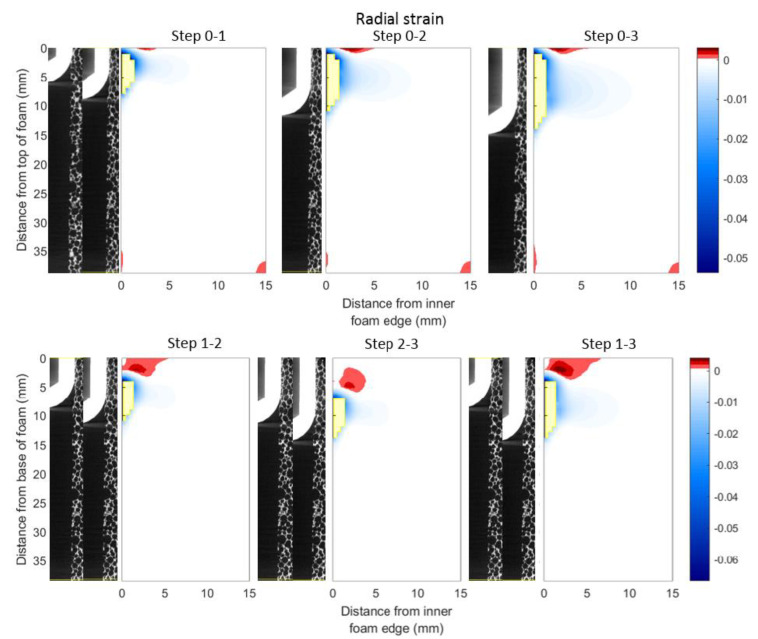
Radial strain fields (averaged circumferentially) for each displacement step relative to reference scan and sequential scans. The top row shows fields calculated from difference with the reference scan (the ‘0’ position); the bottom row shows fields calculated sequentially: between Scans 1 and 2, 2 and 3, and 1 and 3. Note each scan increment corresponds to a target displacement increment of 3.0 mm: Scan 1, 3.0 mm displacement; Scan 2, 6.0 mm displacement; and Scan 3, 9.0 mm displacement. Measurements with correlation coefficients below 0.9 have been excluded and their spatial locations are shown in yellow.

Figure 7 shows the circumferentially averaged implantation strain in the radial direction, comparing each scan to the reference case (top row), and comparing displacement steps (bottom row). Filtering by removing values with correlation coefficients smaller than 0.9 showed a region with degraded measurement accuracy around the implant, although this was reduced for the strain fields calculated sequentially. In the strain fields calculated in comparison to the reference scan, the region of strained material increased with each implantation step, with compressive radial strain propagating over the length of the implant. Peak radial strains were of a similar magnitude for both the strain fields calculated from the reference and those calculated sequentially. In the sequential strain fields, small positive (tensile) strain was observed above the length of new implant interference—indicating strain relief at the top of the analogue bone as implantation progressed.

#### 3.4.2. Hoop Direction

**Figure 8:**
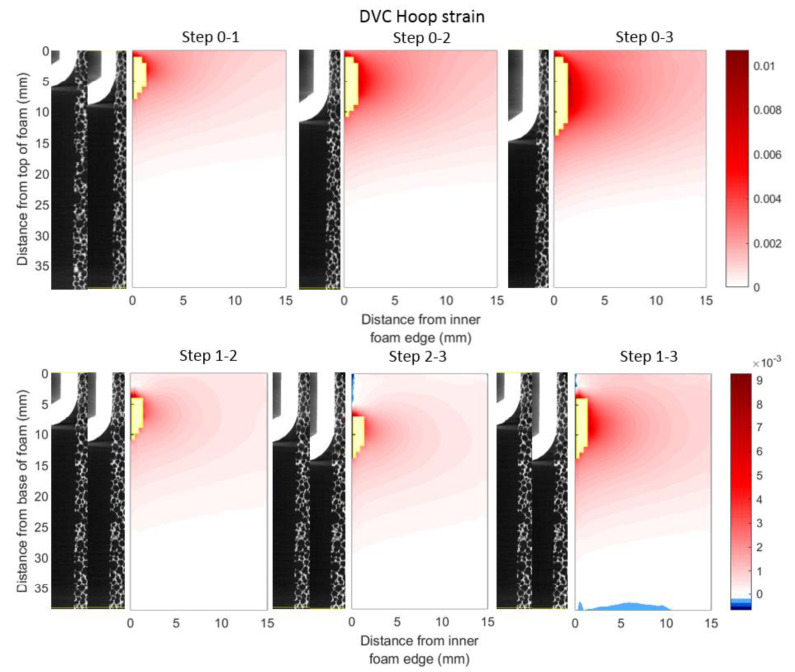
Hoop strain fields (averaged circumferentially) for each displacement step relative to reference scan and sequential scans. Like Figure 7, the top row shows the mean strain measured relative to the reference scan; the bottom row displaying that measured sequentially. Measurements with correlation coefficients below 0.9 have been excluded and their spatial locations are shown in yellow. Note change of strain scale.

Tensile hoop strains were generated next to the implant interface that declined radially, as shown in Figure 8. In all the strain fields calculated compared with the reference scan, a region of high tensile hoop strain was observed ~3 mm from the top of the analogue bone next to the implant. However, a region of diminished strain was present at the top of the analogue bone. The volume of strained material was observed to increase with implant insertion. Between sequential scans, peak tensile hoop strain occurred at the same spatial location as peak compressive radial strain. The region of strained material for the sequential scans was concentrated over the length of new implant interference. In the sequential scans, a small region of compressive strain occurred above the contacting region.

#### 3.4.3. Axial Direction

**Figure 9:**
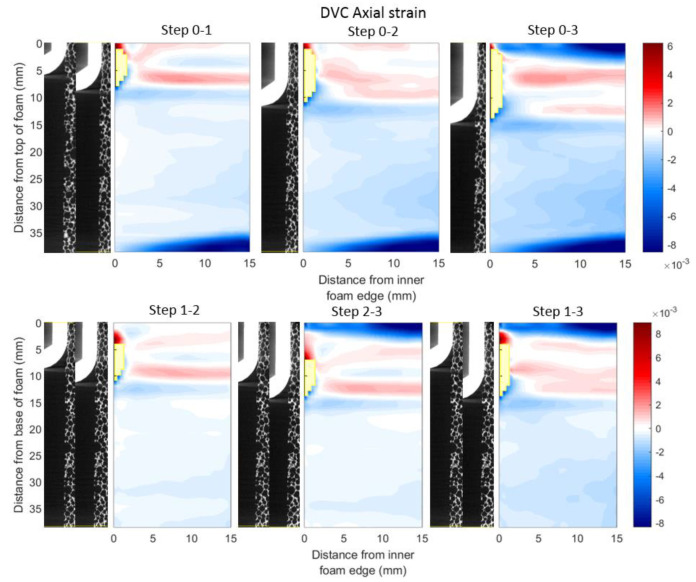
Axial strain fields (averaged circumferentially) for each displacement step relative to reference scan and sequential scans. Like Figure 7 and Figure 8, the top row shows the mean strain measured relative to the reference scan and the bottom displays sequential strains. Measurements with correlation coefficients below 0.9 have been excluded and their spatial locations are shown in yellow.

Figure 9 shows the mean strain fields in the axial direction. In all axial fields, a region of compressive axial strain was present below the position of the implant tip, with a more uniform region of smaller strain propagating toward the base of the analogue bone. However, above the position of the implant tip, regions of positive tensile axial strain were measured. The magnitude of the peak tensile strain was greater than the peak compressive strain. In the fields calculated from the reference, wedges of negative strain were measured at the bottom of the analogue bone for all displacement steps and also at the top for the third displacement step. Similarly, in the sequential scans involving the third displacement scan, a wedge of negative strain was observed at the top of the analogue bone. However, the wedge of negative strain at the bottom of the analogue bone is not observed in any of the sequential strain fields. Additionally, strain banding was observed in the fields, progressing deeper into the analogue bone with each implantation step.

### 3.5. Comparison with Modelling

As the FE results showed similar strain fields for each displacement step, only the fields of the final displacement step (Step 3), compared to the reference scan, are presented.

#### 3.5.1. Radial Direction

**Figure 10:**
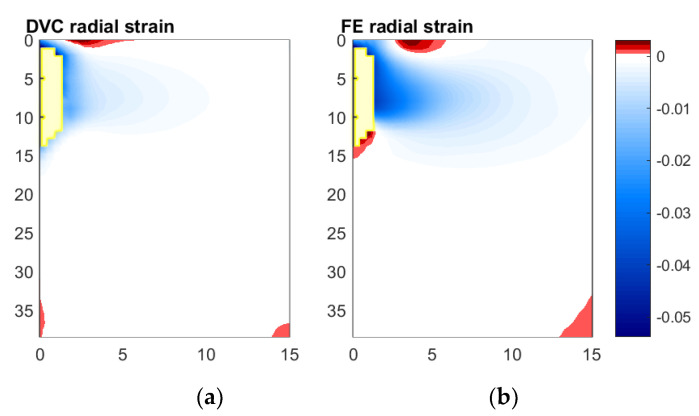
Comparison of radial strain fields measured and predicted, Digital Volume Correlation (**a**) and Finite Element (**b**), for Displacement Step 3 relative to reference scan. Measurements with correlation coefficients below 0.9 have been excluded from both fields, at spatial locations shown in yellow.

Figure 10 compares the radial strain field measured (DVC) with that predicted by the FE model. In the spatial regions where data is available, both the strain fields predicted and measured show compressive radial strain generated over the implant–analogue bone contact region that decays radially. However, the magnitude of the strains measured are substantially smaller than those predicted. Additionally, in the FE strain field, a small region of positive tensile strain is predicted below the implant tip—yet, in the DVC field, this was measured as slightly compressive.

#### 3.5.2. Hoop Direction

**Figure 11:**
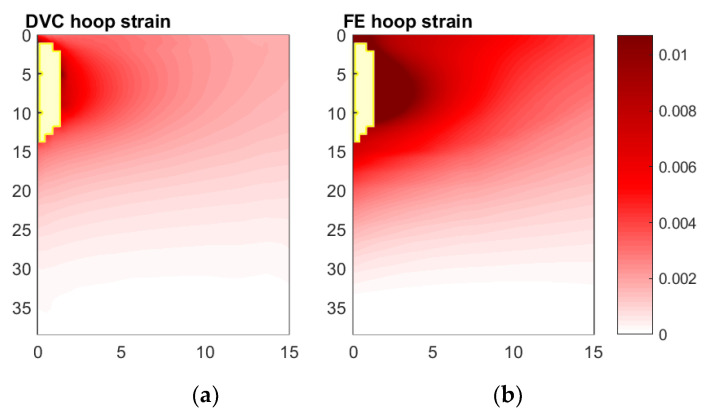
Comparison of hoop strain fields measured and predicted, Digital Volume Correlation (**a**) and Finite Element (**b**), for Displacement Step 3 relative to reference scan. Measurements with correlation coefficients below 0.9 have been excluded from both fields, at spatial locations shown in yellow.

Like the fields of radial strain, the hoop strain fields predicted and measured—shown in Figure 11—were qualitatively similar; for both, tensile hoop strains occurred primarily at the implant–analogue bone interface, decaying radially and ahead of the implant tip. However, again like the fields of radial strain, the magnitude of the hoop strains DVC-measured are substantially smaller than those predicted by FE analysis. Yet, unlike the fields of radial strain, no smaller region exists (in the spatial locations where data is available) where the predicted and measured strain fields contradict each other—the entire field is qualitatively consistent, just lower in magnitude in the measured case.

#### 3.5.3. Axial Direction

**Figure 12:**
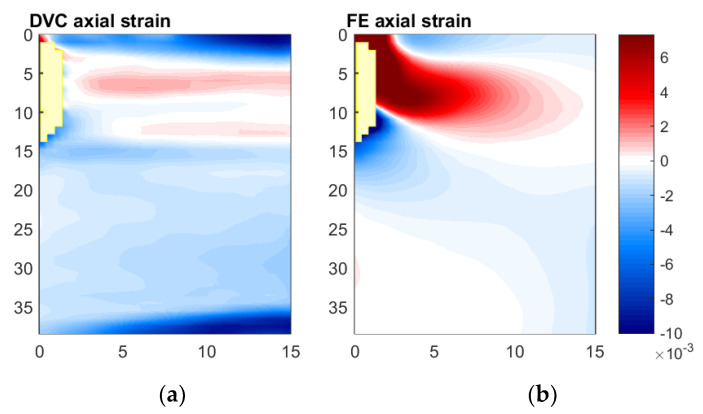
Comparison of axial strain fields measured and predicted, Digital Volume Correlation (**a**) and Finite Element (**b**), for Displacement Step 3 relative to reference scan. Measurements with correlation coefficients below 0.9 have been excluded from both fields, their spatial locations are shown in yellow.

In Figure 12, the axial strain fields predicted and measured are compared. Of all three directions, the axial strain fields show the greatest divergence between that measured using DVC, and that predicted using FE. The same general strain pattern was observed—compressive at the implant tip, followed by a region of tensile propagating diagonally from the top of the analogue bone cavity, but like the radial and hoop strain fields, the magnitude of the strain measured is substantially smaller than that predicted (in the spatial locations where data is available). The point of transition between compressive and tensile also shows a marked difference. In the strain field measured, the compressive axial strain is generated along almost the entire implant–analogue bone interface; tensile strain is only measured close to (within 3 mm of) the top. In the case of the axial strain field predicted, tensile strain is generated over the majority of the implant–analogue bone interface—with the transition between compressive and tensile axial strain close to the implant tip. Finally, the wedge-shaped region of compressive strain observed at the top and bottom of the DVC results, is not observed in the FE predictions.

## 4. Discussion

### 4.1. Experimental Protocol

In presenting this new approach for estimating the implantation strain of a cementless implant, several methodological findings were made:

1. *Identification of suitable DVC parameters.* The subvolume size study indicated a size of 0.97 mm would be a suitable starting point for future DVC experiments involving similar geometries and structures. This was sufficiently sensitive to capture the most important radial and hoop strain signals, with spatial resolution to indicate the continuum-scale strain gradients.
2. *Lack of strain correlation close to implant–analogue bone interface.* Regions of reduced confidence occurred close to the interface, between the implant and analogue bone, where largest strains were generated on implantation. However, regions of reduced strain correlation were not observed during the noise and virtual displacement study. It is likely that in these regions of reduced correlation the degradation was due to high strain caused by localised crushing of the cellular foam, visually observed in the CT scans (Figure 6), from which the analogue bone is made. Future work will need to be mindful of this constraint when cellular material is loaded beyond its elastic response. It is possible that texture on the surface of the implant could aid correlation by introducing a unique pattern.
3. *Rigid body motion and cone-beam artefact*. Several of the axial strain fields contained an unusual feature—a wedge of negative strain—at either the top, the bottom, or both. The cause of this was likely due to a cone-beam artefact in two of the scans. The bottom wedge was generated by a cone-beam artefact on the reference scan, hence being present all the strain fields compared with the reference but none of those calculated sequentially. However, the wedge of negative strain at the top was due to a cone-beam artefact at the top of Scan 3; it was present in all strain fields calculated with the scan at the maximum displacement. The analogue bone’s positioning in the CT scanner changed during the implantation process, moving away from the bottom cone and into the top cone. DVC strain data should not be trusted in regions containing these unavoidable artefacts, so the experimental process could be improved to minimise their effects, for example by translating the scanner stage to correct the rigid body motion of the implant–foam construct.
4. *Scattering by implant tip*. Banding of the strain fields was observed in the axial direction. This is likely to have occurred due to scatter at the implant tip, and where its section thickness changes between its hemispherical tip and hollow cylindrical body. These artefacts were not observed in the radial and hoop strain results, where the strain magnitude was an order of magnitude larger.
5. *Flexibility in custom loading rig*. The difference between the target displacement and that actually achieved was small; rig flexibility resulted in an average achieved implantation of 2.9 mm per step, as opposed to the desired 3.0 mm—a difference of less than 5%. This suggests the custom design was sufficiently stiff for the purposes of this study.

### 4.2. Comparison of Fields Predicted and Measured

Strain fields predicted by FE modelling were found to be qualitatively similar to the DVC measurements, with similar patterns in the radial, hoop, and axial directions. The general features of the radial and hoop strain fields make physical sense: as the implant is displaced into the analogue bone, radial compression is generated and tension imposed circumferentially (tensile hoop strains). This is what would be expected when a slightly larger, stiffer volume is inserted into a softer material with a slightly smaller cavity. Interpretation of the axial strain fields is complicated by the influence of the reference scan. In Scan 0 (reference), the analogue bone is already loaded with 120 N (see Table 2). Therefore, the axial tensile strain observed/predicted when subsequent load steps are compared with the reference represents a reduction in the compression generated locally—not necessarily tensile strain. Viewed from this perspective, the level of observed/predicted axial tension makes physical sense. However, despite the validity of the observations and predictions generally, there are substantial differences between the measured and predicted fields. These differences are instructive:

- *FE results overestimated DVC strain magnitudes in all directions*. The overestimation of the radial, hoop, and axial strains indicates a relaxation of the predicted strain. The likely cause is the local crushing of the cellular structure, which was not incorporated in the bilinear elastoplastic FE constitutive model. When polyurethane foams of the same density have been compressed in displacement control, stress relaxation has been observed in the plateau region of the stress curve [26,36]. Thus, localised crushing near the implant would be expected to reduce load transferred to the rest of the analogue bone, giving a gross reduction in strain. Visual inspection (Figure 6) demonstrated that local crushing was occurring during the tests. The machining of the cavity could have exacerbated the effect: milling of the internal diameter disrupts the cellular structure, and makes it more vulnerable to local collapse under load. Note the size of the foam cells and magnitude of radial displacement due to the interference (0.25 mm) are in the same order of magnitude.
- *Point of transition from compressive to tensile axial strain on implant–analogue bone interface markedly deeper on FE predictions.* In the measured axial strain field, the transition between compressive and tensile axial strain is close to the top of the analogue bone; in the FE simulation, this is much closer to the implant tip. The iteratively tuned coefficients of friction implemented in the FE model may be responsible. All are between 0.030 and 0.036, which is an order of magnitude lower than what would be expected [36,37]. The low tuned coefficient of friction arises from the larger radial compression generated in simulations than in the experiment, as the FE model did not capture localised crushing. Thus, the FE-predicted normal force at the implant–analogue bone interface is too high. To compensate, a small coefficient of friction must be employed to generate an axial load in the simulation to match the experimental measurement. This changes the predicted strain field, with the axial compression at the implant tip having a more significant role in resisting the axial load than frictional shear forces. In contrast, the experimental results suggest axial compression is generated over much of the implant–analogue bone interface. This makes sense, given the much higher coefficient of friction that will be present in reality.
- *Tensile radial strain predicted below the implant tip yet measured as compressive*. Tensile strains (in the analogue bone) ahead of implant arise as an internal reaction to compressive radial strains generated in the region above. The filtering of measurements with correlation coefficients less than 0.9 has obscured this in the field measured using DVC. Thus, the difference between the measured and modelled fields is in the location of the region of tensile radial strain—it is further up in the experimental results. This again occurs as a consequence of the very low coefficients of friction in the simulation. As a result of the dominance of the region close to the implant tip in resisting the axial compression in the FE model, the tensile region of radial strain is shifted further down in the analogue bone.

### 4.3. Implications for Cementless Implants

The differences between the strain fields measured and predicted, and their probable cause, offer insights for cementless implants. Like other recent investigations in the field that use DVC [38], the ability to measure the internal strain allows for analysis not possible with DIC, strain gauges, or extensometers. It is quite possible that cancellous bone would also exhibit a relaxation of the strain generated by implantation, due to localised crushing. If the local disruption of the cellular structure at the interface with the implant is shown to be important, then the size of the bone features relative to the level of interference could be a key determinant in the strain response. For modelling of implantation strains, the results suggest two areas of possible development: use of a constitutive model (for the whole bone) that includes stress relaxation following the initial elastic response; and the use of a different constitutive model for the region of bone local to the surface of the cavity, due to the effect of disrupting the cellular structure.

The insights generated in this work also suggest that further measurement of implantation strains using DVC, combined with FE modelling for improved interpretation, would be valuable. There are several features of the cementless implants that could be investigated with DVC: the effect of taper, asymmetry in the implant geometry, level of interference, and surface finish (roughened or beaded).

## 5. Conclusions

DVC offers a full-field non-contact estimation of implantation strain throughout the bone volume, and provides a rich 3D experimental dataset for comparison with models. To the authors’ knowledge, this methodology had not previously been used to investigate the initial fixation strains generated by a cementless implant. A noise and virtual displacement study identified a suitable subvolume size of 0.97 mm for this work and future studies. Differences between the DVC strain estimates and corresponding FE predictions were revealing. The localised crushing of the analogue bone at the interface with the implant, which occurred experimentally but was not modelled, was identified as the likely cause. This has implications for cementless implant design and its modelling: the size of bone features relative to the level of inference could have a substantial effect on the strain response, and accurate modelling of a cementless implantation may require the incorporation of stress relaxation due to localised crushing—for example, by directly modelling the cellular structure of the bone. The effect of cementless implant design parameters, e.g. surface finish and tapering or asymmetric geometry, would be valuable subjects of further investigation with DVC [39]. 

## Figures and Tables

**Table 1:** Elastic moduli and Poisson’s ratio used in constitutive model for the analogue bone.

Foaming Direction Modulus (*E_z_*)	Transverse Direction Modulus (*E_x_*, *E_y_*)	Shear Modulus (*G_xz_*, *G_yz_*)	Shear Modulus (*G_xy_*)	Foaming Direction Poisson’s Ratio (ν_xy_)	Transverse Direction Poisson’s Ratio (ν_xy_)
505 MPa	357 MPa	187 MPa	137 MPa	0.33	0.28

**Table 2:** Measured displacement, axial load, and coefficient of friction from modelling.

Load Step	Incremental Displacement (mm)	Total Displacement (mm)	Incremental Load (N)	Total Load (N)	Coefficient of Friction
0	1.5	1.5	120	120	0.036
1	2.8	4.3	80	200	0.030
2	2.8	7.1	80	280	0.031
3	3.0	10.1	80	360	0.035
